# Assessing cardiometabolic risk in middle-aged adults using body mass index and waist–height ratio: are two indices better than one? A cross-sectional study

**DOI:** 10.1186/s13098-015-0069-5

**Published:** 2015-09-07

**Authors:** Seán R. Millar, Ivan J. Perry, Catherine M. Phillips

**Affiliations:** Department of Epidemiology and Public Health, HRB Centre for Health and Diet Research, University College Cork, 4th Floor, Western Gateway Building, Western Road, Cork, Ireland

**Keywords:** Body mass index, Waist–height ratio, Cardiometabolic risk, Screening

## Abstract

**Background:**

A novel obesity classification method has been proposed using body mass index (BMI) and waist–height ratio (WHtR) together. However, the utility of this approach is unclear. In this study we compare the metabolic profiles in subjects defined as overweight or obese by both measures. We examine a range of metabolic syndrome features, pro-inflammatory cytokines, acute-phase response proteins, coagulation factors and white blood cell counts to determine whether a combination of both indices more accurately identifies individuals at increased obesity-related cardiometabolic risk.

**Methods:**

This was a cross-sectional study involving a random sample of 1856 men and women aged 46–73 years. Metabolic and anthropometric profiles were assessed. Linear and logistic regression analyses were used to compare lipid, lipoprotein, blood pressure, glycaemic and inflammatory biomarker levels between BMI and WHtR tertiles. Multinomial logistic regression was performed to determine cardiometabolic risk feature associations with BMI and WHtR groupings. Receiver operating characteristic curve analysis was used to evaluate index discriminatory ability.

**Results:**

The combination of BMI and WHtR tertiles identified consistent metabolic variable differences relative to those characterised on the basis of one index. Similarly, odds ratios of having cardiometabolic risk features were noticeably increased in subjects classified as overweight or obese by both measures when compared to study participants categorised by either BMI or WHtR separately. Significant discriminatory improvement was observed for detecting individual cardiometabolic risk features and adverse biomarker levels. In a fully adjusted model, only individuals within the highest tertile for both indices displayed a significant and positive association with pre-diabetes, OR: 3.4 (95 % CI: 1.9, 6.0), P < 0.001.

**Conclusions:**

These data provide evidence that the use of BMI and WHtR together may improve body fat classification. Risk stratification using a composite index may provide a more accurate method for identifying high and low-risk subjects.

## Background

Excess body fat has been shown to be associated with dyslipidaemia, hypertension, insulin resistance, chronic low-grade inflammation and the development of metabolic syndrome (MetS), type 2 diabetes and cardiovascular complications [1–4]. Numerous studies have also demonstrated a high mortality rate in subjects with a body mass index (BMI) ≥30 kg/m^2^ [5]. But because it is a weight-for-height measure, BMI is unable to distinguish between fat and lean mass and elevated BMI may not always indicate increased adiposity or predict cardiometabolic events [6, 7].

Evidence suggests that central obesity is a more important metabolic risk factor and waist circumference (WC) measurement has been recommended as a method for central obesity assessment [8, 9]. However, as WC diagnostic thresholds are different for men and women, and may vary between ethnic groups [10], the practical utility of WC measurement has been questioned [11].

The waist–height ratio (WHtR) (WC divided by height) has been advocated as an alternative surrogate measure of central obesity [12]. As a ratio, this index may circumvent problematic issues relating to gender or population-specific risk cut-points [13, 14]. But results from studies which have compared BMI and WHtR discriminatory abilities have been inconclusive, with some showing WHtR to be only marginally superior to BMI for predicting cardiometabolic outcomes [15–17].

The prevalence of obesity has escalated in many world populations [18]. Thus, there is an increasing need to identify overweight and obese individuals at highest odds of developing cardiometabolic diseases. Recently, a new obesity classification method was proposed, utilising BMI in conjunction with WHtR [14]. Risk stratification using a composite index may provide a more effective method for identifying high and low-risk subjects. This could allow earlier diagnosis, thus attenuating metabolic complications and chronic morbidity development.

The aim of this study was to compare the metabolic profiles in subjects defined as overweight or obese, using BMI and WHtR, in a random sample of 1856 middle-aged men and women. In particular, we examined a range of MetS features, pro-inflammatory cytokines, acute-phase response proteins, coagulation factors and white blood cell (WBC) counts to determine whether a combination of BMI and WHtR more accurately identifies individuals at increased obesity-related cardiometabolic risk.

## Methods

### Study population

The Cork and Kerry Diabetes and Heart Disease Study (Phase II) was a single centre, cross-sectional study conducted between 2010 and 2011. A random sample was recruited from a large primary care centre in Mitchelstown, County Cork, Ireland. The Livinghealth Clinic serves a population of approximately 20,000, with a mix of urban and rural residents. Stratified sampling was employed to recruit equal numbers of men and women from all registered attending patients in the 46–73 year age group. In total, 3807 potential participants were selected from the practice list. Following the exclusion of duplicates, deaths, and subjects incapable of consenting or attending appointment, 3051 were invited to participate in the study and of these, 2047 (49.2 % male) completed the questionnaire and physical examination components of the baseline assessment (response rate: 67.1 %). Details regarding the study design, sampling procedures and methods of data collection have been reported previously [19].

Ethics committee approval conforming to the Declaration of Helsinki was obtained from the Clinical Research Ethics Committee of University College Cork. A letter signed by the contact GP in the clinic was sent out to all selected participants with a reply slip indicating acceptance or refusal. All subjects gave signed informed consent, including permission to use their data for research purposes.

### Clinical and laboratory procedures

All study participants attended the clinic in the morning after an overnight fast and blood samples were taken on arrival. Data on age, gender, morbidity, prescription (Rx) medication use and smoking and alcohol behaviours were gathered through a self-completed General Health Questionnaire (GHQ). Physical activity levels were assessed using the validated International Physical Activity Questionnaire (IPAQ) [20]. Three independent measurements of systolic and diastolic blood pressure (BP) were obtained with the subject in a seated position using an Omron M7 digital sphygmomanometer (Omron Healthcare Co. Ltd., Japan). The mean of the second and third readings was considered to be a subject’s BP.

Triglyceride and high density lipoprotein cholesterol (HDL-C) levels were measured by Cork University Hospital Biochemistry Laboratory on Olympus 5400 biochemistry analysers with Olympus reagents using standardised procedures and fresh samples (Olympus Diagnostica GmbH, Hamburg, Germany). Glucose concentrations were determined using a glucose hexokinase assay (Olympus Life and Material Science Europa Ltd., Lismeehan, Co. Clare, Ireland) and glycated haemoglobin A_1c_ (HbA_1c_) levels were measured in the haematology laboratory on an automated high-pressure liquid chromatography instrument Tosoh G7 [Tosoh HLC-723 (G7), Tosoh Europe N.V, Tessenderlo, Belgium]. Serum insulin, c-reactive protein (CRP), tumour necrosis factor alpha (TNF-α), interleukin 6 (IL-6), adiponectin, leptin, resistin and plasminogen activator inhibitor-1 (PAI-1) were assessed using a biochip array system (Evidence Investigator; Randox Laboratories, UK). Complement component 3 (C3) was measured by immunoturbidimetric assay (RX Daytona; Randox Laboratories). White blood cell counts were determined by flow cytometry technology as part of a full blood count.

### Classification of biochemical and blood pressure measurements

Patients with type 2 diabetes indicated by either HbA_1c_ levels ≥6.5 % (≥48 mmol/mol) or FPG levels ≥7.0 mmol/l, a self-reported physician diagnosis, Rx diabetes medication use, or those who were on insulin therapy, were excluded (N = 184). Lipid, lipoprotein and BP measurements were classified according to National Cholesterol Education Program Adult Treatment Panel III guidelines [21]. Abnormal metabolic risks were defined as high triglycerides ≥1.7 mmol/l and low HDL-C (<1.03 mmol/l in males or <1.29 mmol/l in females). Dyslipidaemia was determined according to both high triglyceride and low HDL-C levels. High BP was classified as systolic BP ≥130 mmHg and/or diastolic BP ≥85 mmHg or Rx anti-hypertensive medication use. The Homeostasis Model Assessment of Insulin Resistance (HOMA-IR) [22] was derived from fasting glucose and insulin concentrations as [(fasting plasma glucose x fasting serum insulin)/22.5] and insulin resistance was defined as a level equal to or above the 75th percentile in the study sample. Having three or more MetS risk features (≥3 metabolic features) was characterised as any combination of the following: high triglycerides, low HDL-C levels, high BP and insulin resistance. Subjects were classified as having pre-diabetes if they had both elevated HbA_1c_ levels ≥5.7 % (≥39 mmol/mol) and impaired fasting plasma glucose levels ≥5.6 mmol/l [23]. As internationally recognised risk cut-points for the examined biomarkers have not been established, we classified inflammation and raised immune activation as a level equal to or above the 75th percentile for each biomarker (C3, CRP, IL-6, TNF-α, leptin, resistin, PAI-1 and WBC) with the exception of adiponectin (equal to or below the 25th percentile).

### Anthropometric variables

The weight and height of each participant were measured to the nearest 0.1 kg and 0.1 cm respectively. Portable electronic Tanita WB-100MA weighing scales (Tanita Corporation, IL, USA) were placed on a firm, flat surface and were calibrated weekly to ensure accuracy. Height was measured using a portable Seca Leicester height/length stadiometer (Seca, Birmingham, UK) and BMI was calculated as weight divided by the square of height. Waist circumference was measured immediately below the lowest rib at the mid-axillary line on bare skin. Subjects were instructed to breathe in, and then out, and to hold their breath while measurement was made to the nearest 0.1 cm using a Seca 200 measuring tape. The WHtR was calculated as WC divided by height. Two independent readings were taken for WC and the mean of the two was used in analysis.

Both BMI and WHtR were divided into equal tertiles. Subjects were categorised on the basis of their BMI or WHtR percentiles as *normal weight* (<33 %), *overweight* (33–66 %) and *obese* (>66 %). In our sample these cut-points corresponded to <26.2, 26.2–29.7, >29.7 for BMI and <0.52, 0.52–0.58, >0.58 for WHtR. The BMI and WHtR groups were combined to form a 5-category variable: (1) *normal weight by both*, (2) *overweight by either*, (3) *overweight by both*, (4) *obese by either* and (5) *obese by both*. Overweight subjects classified as obese by either index were assigned to the higher category. Seven subjects had missing anthropometric values and were excluded from statistical analysis.

### Lifestyle data

Lifestyle variables utilised from the IPAQ [20] and GHQ included physical activity level, smoking status and alcohol use. Self-reported physical activity within the previous 6 months was collapsed into two categories: *high or moderate* (*N* = 1324) and *no physical exercise* (*N* = 312). Subjects were considered to be current smokers if they smoked cigarettes during the recruitment phase of the study (*N* = 257). Alcohol use was assessed by asking study participants how often they consumed alcohol on a monthly or weekly basis, and was dichotomised as follows: ‘never or less than once a month’ and ‘2–4 times monthly’—*occasional drinker* (*N* = 1165), and ‘twice or more weekly’—*regular drinker* (*N* = 614).

### Statistical analysis

Data analysis was conducted using IBM SPSS Statistics Version 20 (IBM Corp., Armonk, NY, USA) and Stata SE Version 13 (Stata Corporation, College Station, TX, USA) for Windows. Descriptive characteristics were examined according to normal weight, overweight and obese defined by BMI and WHtR tertiles. Dichotomous features are presented as percentages and continuous variables are shown as a mean (plus or minus one standard deviation) or a median and interquartile range for skewed data. Linear and logistic regression (adjusting for gender) were used to examine continuous and dichotomous metabolic variable differences between overweight and obese categories. Skewed continuous data were log_10_ transformed. Multinomial logistic regression was performed to determine cardiometabolic risk feature associations with each BMI and WHtR tertile combination. Subjects classified as normal weight by both indices were used as the reference category. All multinomial regression models were adjusted using age, gender, physical activity, smoking status and alcohol use as independent covariates.

The discriminatory ability of BMI, WHtR, and both BMI and WHtR used together, was assessed using receiver operating characteristic curve (ROC) analysis. The area under the curve (AUC) provides a scale from 0.5 to 1.0 (with 0.5 representing random chance and 1.0 indicating perfect discrimination) by which to appraise the capacity of an obesity index to detect a positive result. Three separate analyses were performed. The first analysis assessed each anthropometric measure as a continuous variable. The second analysis explored cardiometabolic risk feature discrimination using index tertiles. A final analysis examined the 5-category BMI/WHtR combination variable used in previous regression models. Significant differences between AUC values were determined. For all analyses, a P value (two-tailed) of less than 0.05 was considered to indicate statistical significance.

## Results

### Descriptive characteristics

The characteristics of the study population were summarised according to BMI and WHtR tertiles (Table 1). A higher tertile level was related to an increased cardiometabolic risk profile as defined by lipid/lipoprotein, BP, glycaemic indicator and biomarker levels, with obese groups showing the highest proportion of cardiometabolic risk factors. In general, cardiometabolic profiles were broadly similar across BMI and WHtR overweight and obese categories, with the percentage of subjects with dyslipidaemia, high BP, insulin resistance, ≥3 metabolic features and pre-diabetes showing little variation according to classification by either index.

**Table 1:** Characteristics of the study population

Feature	Normal weight		Overweight		Obese	
	BMI (N = 619)	WHtR (N = 619)	BMI (N = 618)	WHtR (N = 618)	BMI (N = 619)	WHtR (N = 619)
Male	212 (34.2)	145 (23.4)	346 (56.0)	349 (56.5)	327 (52.8)	391 (63.2)
Age	58 (54, 63)	57 (54, 62)	59 (54, 63)	59 (54, 64)	60 (55, 64)	60 (55, 64)
Weight (kg)	64.6 ± 8.5	65.6 ± 9.4	78.3 ± 9.1	78.6 ± 10.2	92.5 ± 12.6	91.2 ± 13.5
BMI (kg/m^2^)	23.7 ± 1.8	24.3 ± 2.5	27.9 ± 1.0	27.9 ± 2.2	33.3 ± 3.5	32.7 ± 4.0
WC (cm)	79.7 ± 8.9	77.7 ± 7.2	91.8 ± 8.0	92.2 ± 5.8	102.7 ± 10.0	104.3 ± 8.4
WHtR	0.48 ± 0.04	0.47 ± 0.03	0.55 ± 0.04	0.55 ± 0.02	0.62 ± 0.05	0.62 ± 0.04
Triglycerides (mmol/l)	1.0 (0.8, 1.3)	1.0 (0.8, 1.3)	1.2 (0.9, 1.7)	1.2 (0.9, 1.7)	1.4 (1.0, 2.0)	1.5 (1.1, 2.0)
High triglycerides^a^	67 (11.0)	49 (8.0)	147 (24.5)	152 (25.5)	195 (33.3)	208 (35.1)
HDL-C (mmol/l)	1.7 ± 0.4	1.7 ± 0.4	1.4 ± 0.3	1.4 ± 0.3	1.3 ± 0.3	1.3 ± 0.3
Low HDL-C^b^	46 (7.6)	39 (6.4)	74 (12.2)	80 (13.2)	142 (23.8)	143 (24.0)
Dyslipidaemia	12 (2.0)	10 (1.6)	37 (6.1)	37 (6.1)	69 (11.5)	71 (11.9)
Systolic BP (mmHg)	124.9 ± 17.4	124.8 ± 17.2	129.5 ± 15.5	129.2 ± 15.5	133.0 ± 15.9	133.4 ± 16.0
Diastolic BP (mmHg)	77.2 ± 9.6	77.8 ± 9.6	80.7 ± 8.8	80.3 ± 9.4	82.5 ± 9.9	82.3 ± 9.5
High BP^c^	271 (43.8)	263 (42.5)	359 (58.2)	366 (59.3)	471 (76.3)	472 (76.5)
Glucose (mmol/l)	4.8 (4.5, 5.0)	4.7 (4.5, 5.0)	4.9 (4.6, 5.2)	4.9 (4.7, 5.2)	5.1 (4.7, 5.4)	5.1 (4.8, 5.5)
Insulin (µU/ml)	5.3 (3.8, 7.9)	5.3 (3.8, 7.9)	8.4 (5.7, 12.0)	8.3 (5.6, 12.2)	12.9 (8.2, 18.4)	12.9 (8.4, 18.4)
HOMA-IR	1.1 (0.8, 1.7)	1.1 (0.8, 1.7)	1.8 (1.2, 2.7)	1.8 (1.2, 2.7)	2.9 (1.8, 4.3)	2.9 (1.9, 4.3)
Insulin resistance^d^	31 (5.2)	29 (4.9)	121 (20.2)	122 (20.4)	293 (49.4)	294 (49.6)
≥3 metabolic features	16 (2.6)	13 (2.1)	63 (10.2)	59 (9.5)	163 (26.3)	170 (27.5)
HbA_1c_ (%)	5.6 (5.4, 4.8)	5.6 (5.4, 5.8)	5.7 (5.5, 5.8)	5.7 (5.5, 5.9)	5.7 (5.5, 6.0)	5.7 (5.5, 6.0)
Pre-diabetes^e^	27 (4.4)	26 (4.3)	49 (8.1)	41 (6.7)	86 (14.2)	95 (15.7)
C3 (mg/dl)	125.7 ± 22.9	125.2 ± 22.4	134.2 ± 22.7	135.2 ± 22.8	144.5 ± 22.9	144.0 ± 22.8
High C3^f^	79 (13.2)	77 (12.8)	133 (22.0)	137 (22.6)	239 (39.8)	237 (39.6)
CRP (ng/ml)	1.1 (0.8, 1.6)	1.1 (0.8, 1.6)	1.3 (1.0, 1.9)	1.3 (1.0, 2.0)	1.7 (1.2, 3.1)	1.8 (1.2, 3.0)
High CRP^f^	91 (15.1)	85 (14.1)	124 (20.5)	136 (22.4)	236 (39.4)	230 (38.5)
IL-6 (pg/ml)	1.4 (1.0, 2.3)	1.4 (1.0, 2.1)	1.6 (1.2, 2.5)	1.7 (1.2, 2.5)	2.1 (1.5, 3.3)	2.2 (1.5, 3.4)
High IL-6^f^	118 (19.5)	102 (16.9)	126 (20.9)	129 (21.3)	207 (34.5)	220 (36.8)
TNF-α (pg/ml)	5.6 (4.6, 6.9)	5.5 (4.5, 6.6)	5.9 (4.9, 7.2)	5.8 (4.9, 7.1)	6.3 (5.2, 7.5)	6.4 (5.3, 7.7)
High TNF-α^f^	117 (19.4)	110 (18.2)	153 (25.4)	140 (23.1)	181 (30.2)	201 (33.6)
Adiponectin (ng/ml)	6.6 (4.2, 9.8)	6.9 (4.7, 10.2)	4.6 (2.9, 6.9)	4.6 (2.9, 6.9)	4.1 (2.6, 6.3)	3.8 (2.5, 5.5)
Low adiponectin^f^	78 (12.9)	65 (10.8)	176 (29.1)	167 (27.6)	199 (33.2)	221 (36.9)
Leptin (ng/ml)	1.3 (0.6, 2.0)	1.4 (0.8, 2.1)	1.8 (1.0, 2.7)	1.8 (1.0, 2.8)	3.1 (1.9, 5.1)	2.7 (1.6, 4.7)
High leptin^f^	39 (6.5)	67 (11.1)	109 (18.0)	122 (20.1)	303 (50.5)	262 (43.7)
Resistin (ng/ml)	4.8 (3.9, 6.4)	4.9 (3.8, 6.4)	4.9 (3.7, 6.5)	4.9 (3.8, 6.6)	5.3 (4.0, 7.0)	5.2 (4.0, 6.7)
High resistin^f^	133 (22.0)	136 (22.5)	141 (23.3)	152 (25.1)	178 (29.7)	164 (27.4)
PAI-1 (ng/ml)	24.3 ± 10.3	24.0 ± 10.4	28.1 ± 13.6	27.5 ± 11.7	28.7 ± 12.0	29.7 ± 13.7
High PAI-1^f^	100 (16.6)	94 (15.6)	164 (27.2)	161 (26.6)	187 (31.2)	196 (32.8)
WBC (10^9^/l)	5.7 ± 2.4	5.5 ± 1.6	5.8 ± 1.6	5.9 ± 2.3	6.1 ± 1.5	6.3 ± 1.5
High WBC^f^	125 (20.7)	103 (17.0)	149 (24.5)	153 (25.3)	177 (29.7)	195 (32.7)

Mean and ±standard deviation are shown for continuous variables. Age, triglycerides, glucose, insulin, HOMA-IR, HbA_1c_, CRP, IL-6, TNF-α, adiponectin, leptin and resistin are shown as a median (interquartile range). % (in brackets) for dichotomous variables will vary as some variables have missing values

^a^Triglycerides ≥1.7

^b^HDL-C <1.03 (males) or HDL-C <1.29 (females)

^c^Systolic BP ≥130 and/or diastolic BP ≥85 or use of Rx anti-hypertensives

^d^HOMA-IR ≥2.96

^e^Both HbA_1c_ levels ≥5.7 % and fasting plasma glucose levels ≥5.6

^f^Threshold: C3 ≥148; CRP ≥2.25; IL-6 ≥2.72; TNF-α ≥7.2; adiponectin ≤3.1; leptin ≥3.07; resistin ≥6.6; PAI-1 ≥33.66; WBC ≥6.6

### Cardiometabolic profiles according to classification of normal weight, overweight and obese

**Fig. 1:**
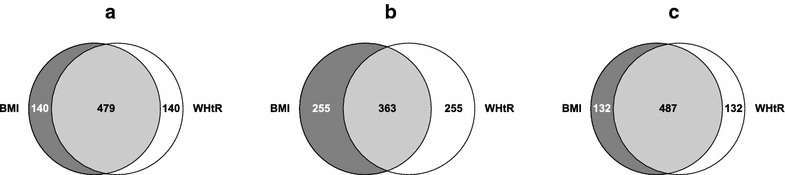
Overlap of normal weight, overweight and obese defined by BMI and WHtR. The figure shows Venn diagrams illustrating overlap of BMI and WHtR tertiles for (**a**) normal weight, (**b**) overweight and (**c**) obese

The levels of agreement between normal weight, overweight and obese tertiles are shown in Fig. 1. Kappa statistics were similar for normal and obese classifications (Kappa: 0.66, SE: 0.02 for normal weight vs. Kappa: 0.68, SE: 0.02 for obese) with marginal overlap between subjects defined as overweight (Kappa: 0.38, SE: 0.02). In both overweight and obese groups (Table 2), the combination of BMI and WHtR tertiles identified consistent and significant metabolic variable differences relative to those characterised on the basis of one index. Subjects that were classified as overweight or obese by both indices displayed higher mean BMI, WC and median triglyceride levels, reduced HDL-C and adiponectin concentrations, and a higher percentage had adverse biomarker levels, insulin resistance, metabolic feature clustering and pre-diabetes.

**Table 2:** Cardiometabolic profiles according to classification of normal weight, overweight and obese defined by either BMI, WHtR or both

Feature	Normal weight by both (N = 479)	Overweight by either (N = 263)	Overweight by both (N = 363)	P value^a^	Obese by either (N = 264)	Obese by both (N = 487)	P value^b^
Male^c^	122 (25.5)	105 (39.9)	231 (63.6)	<0.001	136 (51.5)	291 (59.8)	0.03
Age^d^	58 (54, 62)	58 (54, 65)	59 (54, 63)	0.885	59 (54, 64)	60 (55, 64)	0.15
Weight (kg)^e^	63.1 ± 8.1	71.9 ± 8.0	79.6 ± 9.2	<0.001	81.8 ± 9.5	94.6 ± 12.6	<0.001
BMI (kg/m^2^)^e^	23.3 ± 1.8	26.2 ± 1.5	27.9 ± 1.0	<0.001	29.7 ± 1.9	33.8 ± 3.6	<0.001
WC (cm)^e^	76.6 ± 7.2	85.7 ± 6.7	93.0 ± 5.7	<0.001	95.1 ± 7.2	105.7 ± 8.5	<0.001
WHtR^e^	0.46 ± 0.03	0.52 ± 0.03	0.55 ± 0.02	<0.001	0.57 ± 0.03	0.63 ± 0.05	<0.001
Triglycerides (mmol/l)^e^	0.9 (0.7, 1.2)	1.1 (0.8, 1.5)	1.3 (0.9, 1.7)	0.005	1.3 (1.0, 1.8)	1.5 (1.1, 2.0)	0.034
High triglycerides^e^	36 (7.6)	43 (16.7)	93 (26.6)	0.031	71 (27.7)	166 (35.8)	0.06
HDL-C (mmol/l)^e^	1.7 ± 0.4	1.6 ± 0.4	1.4 ± 0.3	<0.001	1.4 ± 0.3	1.3 ± 0.3	<0.001
Low HDL-C^e^	31 (6.7)	23 (8.8)	46 (12.9)	0.038	39 (15.1)	123 (26.3)	0.001
Dyslipidaemia^e^	7 (1.5)	8 (3.1)	21 (5.9)	0.059	24 (9.3)	58 (12.3)	0.253
Systolic BP (mmHg)^e^	124.2 ± 17.5	126.4 ± 16.2	129.4 ± 14.7	0.027	132.5 ± 16.4	133.4 ± 15.8	0.52
Diastolic BP (mmHg)^e^	77.1 ± 9.6	78.8 ± 9.4	80.5 ± 8.8	0.003	82.2 ± 9.3	82.5 ± 9.8	0.61
High BP^e^	195 (40.7)	134 (51.0)	213 (58.8)	0.069	175 (66.3)	384 (79.2)	<0.001
Glucose (mmol/l)^e^	4.7 (4.5, 5.0)	4.8 (4.6, 5.1)	4.9 (4.7, 5.3)	0.038	4.9 (4.7, 5.2)	5.1 (4.8, 5.5)	<0.001
Insulin (µU/ml)^e^	5.1 (3.7, 7.5)	6.2 (4.3, 9.2)	8.8 (6.0, 12.1)	<0.001	10.2 (6.8, 14.3)	14.0 (9.0, 20.2)	<0.001
HOMA-IR^e^	1.1 (0.8, 1.6)	1.4 (0.9, 2.0)	2.0 (1.3, 2.7)	<0.001	2.2 (1.5, 3.2)	3.2 (2.0, 4.6)	<0.001
Insulin resistance^e^	20 (4.4)	20 (7.8)	72 (20.5)	<0.001	79 (31.1)	254 (54.5)	<0.001
≥3 metabolic features^e^	8 (1.7)	13 (4.9)	35 (9.6)	0.024	39 (14.8)	147 (30.2)	<0.001
HbA_1c_ (%)^e^	5.6 (5.4, 5.8)	5.7 (5.5, 5.9)	5.7 (5.4, 5.8)	0.54	5.7 (5.5, 5.8)	5.8 (5.6, 6.0)	0.002
Pre-diabetes^e^	22 (4.7)	8 (3.1)	31 (8.7)	0.018	21 (8.1)	80 (16.8)	0.002
C3 (mg/dl)^e^	124.0 ± 23.1	130.2 ± 20.4	134.7 ± 23.0	<0.001	139.2 ± 23.2	145.7 ± 22.9	<0.001
High C3^e^	56 (12.1)	42 (16.2)	77 (21.8)	0.013	76 (29.1)	200 (42.6)	<0.001
CRP (ng/ml)^e^	1.0 (0.8, 1.5)	1.3 (1.0, 2.0)	1.3 (1.0, 1.8)	0.976	1.5 (1.1, 2.5)	1.8 (1.2, 3.2)	<0.001
High CRP^e^	60 (12.9)	52 (20.1)	67 (18.9)	0.891	78 (30.1)	194 (41.4)	<0.001
IL-6 (pg/ml)^e^	1.3 (1.0, 2.1)	1.4 (1.1, 2.4)	1.7 (1.2, 2.4)	0.317	1.9 (1.3, 2.9)	2.3 (1.6, 3.5)	0.001
High IL-6^e^	82 (17.6)	49 (18.9)	70 (19.7)	0.99	73 (28.3)	177 (37.7)	0.01
TNF-α (pg/ml)^e^	5.5 (4.6, 6.7)	5.8 (4.6, 6.9)	5.9 (4.9, 7.2)	0.521	5.8 (5.0, 7.3)	6.4 (5.3, 7.0)	0.067
High TNF-α^e^	87 (18.7)	50 (19.4)	89 (25.1)	0.276	68 (26.4)	157 (33.4)	0.047
Adiponectin (ng/ml)^e^	7.0 (4.7, 10.3)	5.9 (3.8, 9.0)	4.2 (2.7, 6.3)	<0.001	4.8 (2.9, 6.7)	3.8 (2.5, 5.5)	0.023
Low adiponectin^e^	48 (10.3)	43 (16.6)	117 (33.0)	0.007	70 (27.0)	175 (37.2)	0.032
Leptin (ng/ml)^e^	1.3 (0.7, 2.0)	1.6 (1.0, 2.4)	1.7 (0.9, 2.7)	0.001	2.3 (1.3, 4.0)	3.2 (1.9, 5.2)	<0.001
High leptin^e^	30 (6.5)	39 (15.1)	56 (15.8)	0.009	87 (33.6)	239 (50.9)	<0.001
Resistin (ng/ml)^e^	4.9 (3.8, 6.4)	4.8 (3.8, 6.7)	4.9 (3.7, 6.4)	0.946	4.9 (4.0, 6.8)	5.3 (4.0, 7.0)	0.274
High resistin^e^	100 (21.5)	66 (25.5)	79 (22.3)	0.452	72 (27.9)	135 (28.7)	0.611
PAI-1 (ng/ml)^e^	23.8 ± 10.2	25.5 ± 10.9	28.1 ± 12.0	0.033	29.3 ± 15.6	29.2 ± 12.1	0.761
High PA1-1^e^	69 (14.8)	52 (20.2)	103 (29.0)	0.046	71 (27.5)	156 (33.2)	0.161
WBC (10^9^/l)^e^	5.5 ± 1.7	5.8 ± 3.0	5.9 ± 1.6	0.92	6.1 ± 1.6	6.2 ± 1.4	0.345
High WBC^e^	84 (17.9)	56 (22.0)	88 (24.6)	0.832	74 (28.7)	149 (31.9)	0.418

Mean and ±standard deviation are shown for continuous variables. Age, triglycerides, glucose, insulin, HOMA-IR, HbA_1c_, CRP, IL-6, TNF-α, adiponectin, leptin and resistin are shown as a median (interquartile range). % (in brackets) for dichotomous variables will vary as some variables have missing values

^a^P value for difference: overweight by either compared to overweight by both

^b^P value for difference: obese by either compared to obese by both

^c^ *x* ^2^ for difference

^d^Mann Whitney U for difference

^e^P value for difference adjusted for gender. Overweight subjects classified as obese by either index were assigned to the higher category

### Associations between cardiometabolic risk features and BMI/WHtR combinations

Table 3 presents results from multinomial logistic regression models examining each BMI and WHtR tertile combination. A clear dose–response association was noted, with odds ratios of having cardiometabolic risk features being noticeably increased in subjects classified by both indices. In univariate analysis (not shown), odds ratios of having pre-diabetes were 0.6 (95 % CI: 0.3, 1.5), 1.9 (95 % CI: 1.1, 3.4), 1.8 (95 % CI: 1.0, 3.3) and 4.1 (95 % CI: 2.5, 6.7) for subjects categorised as *overweight by either*, *overweight by both*, *obese by either* and *obese by both* measures respectively. In a fully adjusted model, only patients within the highest BMI and WHtR tertile displayed a significant and positive association with pre-diabetes defined by both HbA_1c_ and fasting plasma glucose levels, OR: 3.4 (95 % CI: 1.9, 6.0), P < 0.001.

**Table 3:** Odds ratios (95 % CI) of having cardiometabolic risk features according to classification of overweight and obese

Feature	Odds ratios (95 % CI)^a^							
	Overweight compared to normal weight				Obese compared to normal weight			
	Either BMI or WHtR	P value	Both BMI and WHtR	P value	Either BMI or WHtR	P value	Both BMI and WHtR	P value
High triglycerides	2.1 (1.3, 3.5)	0.003	3.5 (2.3, 5.4)	<0.001	3.4 (2.1, 5.5)	<0.001	5.6 (3.7, 8.6)	<0.001
Low HDL-C	1.4 (0.8, 2.5)	0.3	2.1 (1.3, 3.7)	0.005	2.2 (1.2, 3.8)	0.008	5.8 (3.6, 9.2)	<0.001
Dyslipidaemia	1.8 (0.6, 5.3)	0.263	3.8 (1.5, 9.3)	0.004	4.6 (1.9, 11.6)	0.001	8.6 (3.7, 19.6)	<0.001
High BP	1.5 (1.1, 2.1)	0.02	2.1 (1.5, 2.8)	<0.001	3.0 (2.1, 4.2)	<0.001	5.7 (4.1, 7.9)	<0.001
Insulin resistance	1.8 (0.9, 3.7)	0.083	5.4 (3.1, 9.6)	<0.001	9.5 (5.3, 16.8)	<0.001	26.6 (15.5, 45.7)	<0.001
≥3 metabolic features	2.6 (1.0, 6.6)	0.043	5.4 (2.4, 12.0)	<0.001	7.8 (3.5, 17.5)	<0.001	22.2 (10.5, 47.0)	<0.001
Pre-diabetes	0.6 (0.2, 1.4)	0.227	1.6 (0.9, 3.1)	0.142	1.6 (0.8, 3.2)	0.218	3.4 (1.9, 6.0)	<0.001
High C3	1.3 (0.8, 2.1)	0.260	2.8 (1.9, 4.3)	<0.001	3.3 (2.1, 5.0)	<0.001	7.9 (5.4, 11.6)	<0.001
High CRP	1.6 (1.0, 2.6)	0.032	1.8 (1.2, 2.7)	0.007	3.6 (2.4, 5.5)	<0.001	6.1 (4.2, 8.9)	<0.001
High IL-6	1.0 (0.7, 1.6)	0.897	1.1 (0.8, 1.7)	0.541	1.7 (1.2, 2.6)	0.008	2.7 (1.9, 3.8)	<0.001
High TNF-α	1.1 (0.7, 1.6)	0.738	1.4 (1.0, 2.0)	0.089	1.2 (0.8, 1.9)	0.323	2.2 (1.5, 3.1)	<0.001
Low adiponectin	1.4 (0.8, 2.3)	0.204	2.7 (1.8, 4.2)	<0.001	2.6 (1.6, 4.2)	<0.001	3.9 (2.6, 6.0)	<0.001
High leptin	3.6 (2.1, 6.3)	<0.001	5.9 (3.5, 9.9)	<0.001	15.7 (9.3, 26.6)	<0.001	46.6 (27.9, 77.6)	<0.001
High resistin	1.3 (0.9, 1.9)	0.205	1.3 (0.9, 1.9)	0.194	1.5 (1.0, 2.2)	0.046	1.8 (1.2, 2.5)	0.001
High PAI-1	1.2 (0.8, 1.8)	0.460	2.0 (1.3, 2.9)	<0.001	1.9 (1.3, 2.9)	0.002	2.7 (1.9, 3.9)	<0.001
High WBC	1.5 (1.0, 2.4)	0.073	1.9 (1.2, 2.9)	0.003	2.7 (1.7, 4.2)	<0.001	3.2 (2.2, 4.8)	<0.001

^a^Multinomial logistic regression, reference category: normal weight by both BMI and WHtR. Overweight subjects classified as obese by either index were assigned to the higher category. All models adjusted for age, gender, physical activity, smoking and alcohol use

### Discrimination of cardiometabolic risk features

In ROC analysis (Table 4), when used as a continuous variable, significantly higher AUC values for WHtR were found to discriminate high triglycerides, ≥3 metabolic features, elevated C3 and WBC levels when compared to BMI. BMI displayed a significantly higher AUC for detecting increased leptin levels compared to WHtR. A combination of both measures displayed significantly higher discriminatory accuracy for high triglycerides, metabolic feature clustering, C3 and CRP compared to BMI, and for leptin compared to WHtR. Significant improvement for detecting insulin resistance and high WBC levels were noted compared to when either BMI or WHtR were used independently.

**Table 4:** Area under the receiver operating characteristic curve values (95 % CI) for index models to discriminate cardiometabolic risk features

Feature	As a continuous variable						As a categorical variable (tertiles)				Overweight and obese by either or both^a^	
	BMI alone		WHtR alone		Both BMI and WHtR		BMI alone		WHtR alone			
	AUC	95 % CI	AUC	95 % CI	AUC	95 % CI	AUC	95 % CI	AUC	95 % CI	AUC	95 % CI
High triglycerides	0.68	0.65, 0.71	0.71^b^	0.68, 0.73	0.71^b^	0.68, 0.74	0.68	0.65, 0.71	0.70^d^	0.67, 0.73	0.70^d^	0.67, 0.73
Low HDL-C	0.67	0.63, 0.70	0.68	0.65, 0.72	0.68	0.65, 0.71	0.66	0.62, 0.69	0.67	0.64, 0.71	0.68^d^	0.64, 0.71
Dyslipidaemia	0.68	0.64, 0.73	0.70	0.65, 0.74	0.70	0.65, 0.74	0.69	0.64, 0.73	0.69	0.65, 0.73	0.70	0.66, 0.74
High BP	0.70	0.67, 0.72	0.70	0.67, 0.72	0.70	0.68, 0.72	0.69	0.67, 0.72	0.69	0.67, 0.71	0.70	0.67, 0.72
Insulin resistance	0.80	0.78, 0.82	0.80	0.78, 0.83	0.81^b,c^	0.79, 0.83	0.78	0.76, 0.80	0.77	0.75, 0.80	0.79^d,e^	0.77, 0.82
≥3 metabolic features	0.76	0.73, 0.79	0.78^b^	0.75, 0.81	0.78^b^	0.75, 0.81	0.75	0.72, 0.78	0.75	0.72, 0.78	0.77^d,e^	0.74, 0.80
Pre-diabetes	0.70	0.66, 0.74	0.70	0.66, 0.74	0.70	0.66, 0.74	0.67	0.63, 0.71	0.68	0.64, 0.72	0.69^d^	0.65, 0.73
High C3	0.70	0.67, 0.73	0.72^b^	0.69, 0.75	0.72^b^	0.69, 0.75	0.69	0.66, 0.71	0.70	0.67, 0.73	0.71^d^	0.68, 0.73
High CRP	0.69	0.66, 0.72	0.69	0.67, 0.72	0.70^b^	0.67, 0.72	0.67	0.64, 0.70	0.68	0.65, 0.71	0.69^d^	0.66, 0.72
High IL-6	0.66	0.63, 0.69	0.67	0.64, 0.69	0.67	0.64, 0.69	0.65	0.62, 0.68	0.66	0.63, 0.69	0.66^d^	0.63, 0.69
High TNF-α	0.63	0.60, 0.66	0.63	0.60, 0.66	0.63	0.60, 0.66	0.62	0.59, 0.65	0.63	0.61, 0.66	0.63^d^	0.60, 0.66
Low adiponectin	0.79	0.77, 0.81	0.79	0.77, 0.81	0.79	0.77, 0.81	0.79	0.77, 0.81	0.79	0.76, 0.81	0.79	0.77, 0.81
High leptin	0.86^c^	0.84, 0.87	0.84	0.82, 0.86	0.86^c^	0.84, 0.88	0.84^e^	0.82, 0.86	0.81	0.79, 0.83	0.84^e^	0.82, 0.86
High resistin	0.57	0.54, 0.60	0.56	0.53, 0.59	0.57	0.54, 0.60	0.57	0.54, 0.60	0.56	0.53, 0.59	0.56	0.53, 0.60
High PAI-1	0.61	0.58, 0.64	0.62	0.59, 0.65	0.62	0.59, 0.65	0.62	0.59, 0.64	0.62	0.59, 0.65	0.62	0.59, 0.65
High WBC	0.59	0.56, 0.62	0.61^b^	0.58, 0.64	0.63^b,c^	0.60, 0.66	0.58	0.55, 0.61	0.60^d^	0.57, 0.63	0.59^d^	0.56, 0.62

All models include age and gender

^a^5-category variable: 1 normal weight by both, 2 overweight by either, 3 overweight by both, 4 obese by either and 5 obese by both. Overweight subjects classified as obese by either index were assigned to the higher category

^b^P < 0.05 compared to BMI (continuous)

^c^P < 0.05 compared to WHtR (continuous)

^d^P < 0.05 compared to BMI (categorical)

^e^P < 0.05 compared to WHtR (categorical)

When indices were examined as tertiles, significant differences between BMI and WHtR remained for discriminating high triglyceride, leptin and WBC concentrations. The BMI/WHtR 5-category variable was a significantly better discriminator of high triglycerides, low HDL-C, pre-diabetes, high C3, CRP, IL-6, TNF-α and WBC levels compared to BMI, and of leptin compared to WHtR. Significantly higher AUC values for detecting insulin resistance and ≥3 metabolic features were also found compared to when either measure were used alone.

## Discussion

The aim of this study was to determine whether risk stratification using BMI and WHtR together more accurately identifies individuals at increased obesity-related cardiometabolic risk. Our findings indicate that both measures classify different subjects, particularly within the overweight range. These results also demonstrate that individuals defined as overweight or obese, by both BMI and WHtR, exhibit different cardiometabolic profiles compared to subjects categorised by either index separately. Participants identified by both measures demonstrated stronger associations with individual cardiometabolic risk factors, metabolic feature clustering and displayed a more pro-inflammatory, pro-antherogenic and insulin resistant profile. Use of both indices also significantly improved discrimination of cardiometabolic risk features. These results suggest that joint use of BMI and WHtR may be clinically useful as a method to detect individuals at risk of cardiometabolic disorders.

Although it is straightforward to assess, and easy to calculate, limitations regarding the use of BMI as a sole method for adiposity appraisal have been widely acknowledged [7, 11]. Though frequently employed within epidemiological research and healthcare practice, BMI does not discriminate between fat and lean body mass, therefore persons of short stature or muscular build may be misidentified [24]. Recent research has indicated that general obesity categorisation based on BMI might be inadequate [25]. Importantly, the finding that approximately half of obese subjects are metabolically healthy when classified using dual-energy X-ray absorptiometry-derived body fat percentage, compared to approximately one-third by BMI [26], signals that caution should be exercised with regard to how obesity is defined [24].

Compared with BMI, WC is thought to be more strongly correlated with visceral adipose tissue (VAT) which has been shown to be associated with increased risk of dyslipidaemia, hypertension and type 2 diabetes [9, 27, 28]. Though the exact mechanism of association between VAT and metabolic risk is still poorly understood, research has implied that fatty acids released from VAT drain into the liver and skeletal muscle, causing metabolic dysfunction within these organs [29]. Adipokines secreted from VAT may also contribute to cardiometabolic disease through inflammation of vascular tissue [8, 30]. Although WC measurement has been recommended as a method for VAT and cardiometabolic risk assessment, controversy exists regarding its clinical efficacy. In particular, the need for gender and ethnic-specific risk cut-points, and the fact that WC does not take whole body fat distribution into account, indicate constraints regarding its practical application and usefulness within a clinical setting [11].

The WHtR is potentially advantageous as it may not require conversion to gender or population-specific cut-offs or percentiles [13]. It has been previously suggested that a WHtR ≥0.5 may serve as a useful boundary for increased cardiometabolic risk, with a WHtR ≥0.6 threshold indicating substantially increased risk [14]. Additionally, it has been shown that height has an inverse association with cardiovascular disease mortality and total mortality [31, 32], indicating that its use within an adiposity variable may be clinically important. In a recent meta-analysis of 31 prospective or cross-sectional studies, Ashwell et al. demonstrated WHtR to be a better discriminator of hypertension, metabolic syndrome, type 2 diabetes and cardiovascular disease when compared to BMI [15]. Pooled results showed that WHtR improved discrimination of all outcomes by 3–4 %. However, other studies have suggested that differences in predictive abilities are minimal, and have questioned the measurement of height in addition to WC [17].

The results from our research suggest that both BMI and WHtR provide important and independent information, and that joint measurement may help refine body fat risk classification. Within our sample it was noted that participants who were categorised as overweight on the basis of one index also displayed an increased cardiometabolic risk profile. As a percentage of these individuals might be considered normal weight if either measure were used alone, these results indicate that use of both indices may provide a more sensitive method for detecting patients at increased cardiometabolic risk. We also observed noticeably strong associations with cardiometabolic risk factors in subjects who were classified as overweight or obese by both BMI and WHtR together. This suggests that joint measurement may equally provide a more specific procedure for identifying high-risk subjects within overweight and obese categories. In particular, patients within the highest tertile for both indices were at a significantly higher risk compared to other obese subjects. In addition, a significant association with pre-diabetes was only observed within this tertile after adjustment for other risk factors. This might imply that the relationship between obesity and diabetes is better indicated at this mode and level of adiposity.

Nevertheless, it was also noted that subjects classified by both indices were, on average, more overweight or obese, and thus would probably be identified if either BMI or WHtR were used alone. Additionally, discriminatory improvements for detecting individual cardiometabolic features were modest. However, cardiometabolic diseases are multifactorial, as it has been shown that subjects with a combination of features are at higher risk of cardiometabolic events [33, 34]. Also, a degree of measurement error is to be expected during any anthropometric assessment. We have recently shown that using BMI and WHtR together significantly improves discrimination of type 2 diabetes [30]. Cut-points on the ROC demonstrated greater sensitivity and specificity than when either index were used independently. As the sum of risk factors may be greater than the individual parts for predicting cardiometabolic events, and as measurement error may limit the minimal detectable difference in a cardiometabolic risk parameter [35], it could be that these findings are due to the greater measurement accuracy that joint BMI and WHtR assessment may provide.

Although it is hoped that public health programs may eventually reduce the prevalence of obesity-related metabolic disorders, current strategies to combat obesity are failing as overweight and obesity rates continue to increase worldwide [18]. As a percentage of obese subjects are considered to be metabolically healthy [24], there is an increasing need for cheap and non-invasive methods to detect overweight and obese individuals at highest odds of developing cardiometabolic diseases. In previous research we have shown that assessing both bioelectrical impedance-derived body fat percentage and BMI may help to discriminate individuals at greater cardiometabolic risk than BMI alone [36]. Those identified using both tools had a more metabolically unhealthy profile and were non-responsive to dietary changes. These findings suggest that stratification of obese individuals, based on their metabolic health phenotype, could be important in the early identification of those who should be prioritised for pharmacological and lifestyle interventions [24]. Joint use of BMI and WHtR may provide a convenient and inexpensive means for risk stratification. Such a method might be useful in resource-poor settings where blood sampling is unavailable, or in populations without regular access to primary health services.

As far as we are aware, our study is the first to comprehensively examine the joint use of BMI and WHtR in a middle-aged European population. Strengths include a high participation rate, the use of questionnaires to assess lifestyle behaviours and inclusion of a wide range of metabolic variables to define cardiometabolic health. Our findings are of potential public health and clinical significance in terms of screening and the use of stratification based on obesity assessment as a method for determining cardiometabolic risk.

Notwithstanding these strengths, methodological limitations should be considered when examining results from this study. Given the modest number of outcomes within our sample, we did not stratify by gender in analysis. Although some studies have implied heterogeneous relationships between measures of adiposity and cardiometabolic outcomes relating to gender [11], previous work by our group has suggested that these may be explained by sex differences in obesity prevalence [30]. In addition, recommended risk cut-points for BMI and WHtR are the same for men and women, and the gender variable was accounted for in statistical analyses.

Also of concern is that we did not use established obesity index cut-offs and that our data were cross-sectional, as this precludes examination of temporal relationships. Although World Health Organisation cut-points for BMI are commonly used [5], and thresholds for WHtR have been recommended [14, 37], for the purposes of this research it was necessary to place both variables on the same scale. Future studies, utilising longitudinal data, will be needed to evaluate the applicability, validity and reliability of joint measurement [14] using established and recommended diagnostic cut-points. In particular, it will be necessary to determine whether risk stratification, using both BMI and WHtR, is clinically useful and superior to currently recommended BMI classification [38].

Finally, our data were derived from a single primary care based sample which may not be representative of the source population. However, Ireland presents a generally ethnically homogeneous group [39]. Thus, the relationships we observed are likely to be similar in other middle-aged Irish adults. In addition, random sampling of subjects and the use of validated methods for data collection ensured internal sample validity and the results from this research may be generalisable to a similar middle-aged Caucasian-European population.

## Conclusions

In summary, our findings reveal that cardiometabolic risk profiles in individuals defined as overweight or obese, by both BMI and WHtR, are significantly increased when compared to subjects categorised by either index separately. Use of both measures also improved discrimination of individual cardiometabolic risk factors and identified a subset of at-risk individuals who might otherwise be missed. Although assessment of WC, in addition to BMI, competes for the limited time available during patient appraisal within clinical practice [9], obtaining two measurements (one for general obesity, and one for central obesity) does not entail any extra cost [14]. In light of the increasing prevalence of cardiometabolic diseases worldwide [40], effective methods that help to identify subjects at greatest risk are needed. Risk stratification utilising BMI and WHtR together may provide a simple, cost-effective and more accurate technique for predicting obesity-related cardiometabolic events. Earlier identification of individuals at risk could enable earlier targeted interventions or therapies, thus attenuating development of cardiovascular complications.

## Abbreviations

AUC: area under the curve
BMI: body mass index
BP: blood pressure
CI: confidence interval
C3: complement component 3
CRP: c-reactive protein
GHQ: general health questionnaire
HbA_1c_: glycated haemoglobin A_1c_
HDL-C: high density lipoprotein cholesterol
IL-6: interleukin 6
IPAQ: international physical activity questionnaire
MetS: metabolic syndrome
OR: odds ratio
PAI-1: plasminogen activator inhibitor-1
ROC: receiver operating characteristic curve
Rx: prescription
TNF-α: tumour necrosis factor alpha
VAT: visceral adipose tissue
WBC: white blood cell
WC: waist circumference
WHtR: waist–height ratio
